# The Polycystic Ovary Syndrome: An update on metabolic and hormonal mechanisms

**Published:** 2015

**Authors:** R Dumitrescu, C Mehedintu, I Briceag, VL Purcarea, D Hudita

**Affiliations:** * “Carol Davila” University of Medicine and Pharmacy, Bucharest, Romania; ** Gynecology Department, “Nicolae Malaxa Hospital”, Bucharest, Romania; *** Gynecology Department, “Dr. I. Cantacuzino Hospital”, Bucharest, Romania

**Keywords:** PCOs, hyperandrogenism, infertility, insulin resistance, criteria

## Abstract

Polycystic ovary syndrome (PCOs) is a public health important disease, affecting one in five women at reproductive age. The clinical implications include reproductive, metabolic and psychological features. This article reviews the literature data related to the new metabolic and hormonal mechanisms in PCOs.

Recognizing the real diagnostic of PCOs, using the right criteria, is a challenge in current practice.

PCOs is now recognized as one of the most common endocrinopathy in women at reproductive age with a prevalence of 4–10% for the NICHD defined form [**1**-**4**]. These prevalence estimates the use of the NICHD criteria for PCOs, which are remarkably consistent across racial and ethnic groups [**1**,**2**,**4**-**6**].

Polycystic ovary syndrome (PCOs) is an important metabolic as well as reproductive disorder conferring a substantially increased risk for type 2 diabetes. Affected women have marked insulin resistance, independent of obesity. The obese women with PCOs are insulin resistant, but some groups of lean affected women may have normal insulin sensitivity. There is a post-binding defect in receptor signaling due to increased receptor and insulin receptor substrate-1 serine phosphorylation that selectively affects metabolic, but not mitogenic pathways in classic insulin target tissues and in the ovary. 

The constitutive activation of serine kinases in the MAPK-ERK pathway may contribute to the resistance to the insulin's metabolic actions in the skeletal muscle. Insulin functions as a co-gonadotropin through its cognate receptor to modulate ovarian steroidogenesis. The genetic disruption of insulin signaling in the brain has indicated that this pathway is important for the ovulation and body weight regulation. These insights have been directly translated into a novel therapy for PCOs with insulin-sensitizing drugs. 

**Insulin resistance and PCOs**

In the 19th century, the ovarian wedge resection became a recommended therapy [**7**], although Stein and Leventhal [**8**] first reported that the clinical features of menstrual regularity and infertility could be improved by the removal of portions of both ovaries. As a result, the constellation of enlarged, sclerocystic ovaries frequently associated with hirsutism, menstrual irregularity, obesity, and infertility became known as the Stein-Leventhal syndrome [**7**,**9**]. In recent decades, PCOs has become the preferred terminology [**7**,**10**]. In 1980, Burghenet al. [**11**] reported that women with PCOs had increased insulin responses during oral glucose tolerance testing that were not accounted for by obesity. Furthermore, women with typical PCOs had acanthosis nigricans, raising the possibility of being insulin resistant, similar to women with the rare syndromes of extreme insulin resistance [**12**,**13**]. These observations launched a new field of study on the mechanisms for the association between insulin resistance and PCOs.

**Insulin resistance and concomitant hyperinsulinemia**

Insulin resistance and concomitant hyperinsulinemia are frequently found in obese PCOs women [**14**,**15**]. Increased insulin resistance causes hyperglycemia leading to hyperinsulinemia and it amplifies LH action on theca cells and again increases in androgen level [**16**,**17**]. Hyperinsulinemia, insulin resistance and an increase in androgen production are all linked together in PCOs patients. The patients with insulin resistance are often resistant to ovulation induction [**15**,**16**].

**Insulin resistance and abnormal glucose metabolism**

Insulin resistance occurs in around 50% to 80% of women with PCOs, primarily in the more severe NIH diagnosed PCOs and in those who are overweight. Lean women and milder Rotterdam diagnosed PCOs appear to have less severe insulin resistance. A full discussion of the complex mechanisms involved in insulin resistance, hyperinsulinemia, DM2 and CVD is beyond the purpose of this review. The mechanisms involved in insulin resistance are likely to be complex with genetic and environmental contributors. Specific abnormalities of insulin metabolism identified in PCOs include reductions in secretion reduced hepatic extraction, impaired suppression of hepatic gluconeogenesis and abnormalities in insulin receptor signaling. Interestingly, there is a paradoxical expression of insulin resistance in PCOs, whereby insulin-stimulated androgen production persists while its role in glucose metabolism is impaired. Therefore, insulin resistance in PCOs results in hyperinsulinemia with its associated diverse and complex effects on regulating lipid metabolism, protein synthesis and modulation of androgen production. The cause of insulin resistance is likewise complex and multifactorial with genetic and environmental contributors. Lean women with PCOs often, but not always have abnormalities of insulin secretion and action compared to weight-matched control subjects. While a woman with PCOs is overweight, she may also demonstrate extrinsic insulin resistance associated with adiposity, which is potentially mechanistically distinct from the insulin resistance present in lean women with PCOs. In women with insulin resistance and PCOs, only a subgroup develops coexistent pancreatic insufficiency with β cell failure and go on to DM2. In this setting, insulin output cannot overcome resistance and hyperglycemia development. Women with PCOs are at increased risk of developing IGT and DM2 with prevalence rates of 31.3% and 7.5%, respectively, compared to 14% for IGT and 0% for DM2 in age-matched and weight-matched non-PCOs control women.

Women with PCOs also develop abnormal glucose metabolism at a younger age and may demonstrate a more rapid conversion from IGT to DM2.

Impaired fasting glucose is a poor predictor of IGT in women in general and in PCOs. Hence, the ESHRE/ ASRM-sponsored PCOs Consensus Workshop Group recommend an oral glucose tolerance test in all overweight women with PCOs. Furthermore, emerging data shows an increased risk of metabolic complications in the first-degree family members of women with PCOs.

**Investigations and assessment in PCOs**

Key investigations include prolactin and thyroid stimulating hormone to exclude other disorders and testosterone, SHBG and free androgen index to assess androgen status.

They also include a pelvic ultrasound for ovarian morphology and endometrial thickness. An oral glucose tolerance test (rather than fasting glucose) and lipid profiles are appropriate in all women at diagnosis and 1 to 2 yearly after this, where women are overweight or have an increased risk of DM2 (for example, family history of DM2 in first-degree relatives, increased age or high-risk ethnic group). As noted, insulin levels should not be measured in clinical practice because of assay variability and inaccuracy. Metabolic syndrome and abnormal glucose metabolism best reflect insulin resistance in this population.

**A. Clinical features**

Approximately 60% of women with PCOs are hirsute, the most common clinical sign of hyperandrogenemia [**18**]. Acne and androgenic alopecia are other clinical signs of hyperandrogenemia [**19**–**26**]. It is evident on clinical examination in a substantial percentage of obese women with PCOs as well as in some lean affected women. Many lean women with PCOs also show histological evidence of acanthosis nigricans [**27**]. Its severity is directly correlated with the degree of insulin resistance [**27**,**28**].

Oligomenorrhea is defined as menstrual cycles that are longer than 35 d (usually fewer than eight cycles per year) and is a sign of anovulatory cycles [**18**]. However, regular menstrual cycles do not exclude chronic anovulation, especially in women with clinical signs of androgen excess [**18**].

**B. Biochemical profile in PCOs**

Sex hormones

Hyperandrogenemia is the biochemical hallmark of PCOs [**18**].

Elevated luteinizing hormone and insulin synergistically increase androgen production. Insulin resistance leads to hyperinsulinemia, reduces SHBG and raises free circulating testosterone and together, hyperandrogenism and hyperinsulinemia impairs ovarian follicle development. The clinical hyperandrogenism primarily includes hirsutism, acne and male pattern alopecia. Hirsutism is defined in females as male type terminal hair growth and distribution. PCOs is a common cause of hirsutism occurring in approximately 60% of the cases, however, this varies according to race and degree of obesity. Hirsutism should be assessed with a standardized scoring system (Ferriman-Gallwey score).

The other features of hyperandrogenism include virilization, which, especially if presenting with a clitoromegaly and rapid onset, requires the exclusion of other causes including adrenal or ovarian androgen-secreting tumors.

Elevated circulating androgen levels are observed in 80–90% of women with oligomenorrhea. Elevated levels of free T account for the vast majority of abnormal findings in the laboratory examination. This finding reflects the fact that SHBG levels are typically decreased in PCOs due to the effects of T and insulin to decrease hepatic production of SHBG.

The measurement of total and free T levels is constrained by the available assay methods. Assays for total T lack precision and sensitivity in the female T range, including T levels typical of PCOs. The accurate measurement of free T by equilibrium dialysis is technically challenging and costly, whereas direct measurement of free T is inaccurate. The measurements of total T by RIA or liquid chromatography-mass spectrometry in a specialized endocrine laboratory are currently the best available methodologies. Free and biologically available T can be calculated from the concentrations of total T, SHBG, and albumin by using the affinity constants of T for these molecules. 

Free androgen index measurements are generally recommended, derived in the lab from SHBG and total testosterone measurements.

It is unclear whether the concurrent measurement of androstenedione increases the diagnosis of hyperandrogenemia. Approximately 25% of women with PCOs will have elevated levels of dehydroepiandrosterone sulfate (DHEAS), which may be the sole abnormality in circulating androgens in approximately 10% of these women.

Although the ovaries are the main source of increased androgens in PCOs, adrenal androgen excess is a common feature of the syndrome. 

Dehydroepiandrosterone sulfate (DHEAS) and androstenedione are not routinely recommended in PCOs.

Women with PCOs demonstrate an increased secretion of adrenocortical precursor steroids basally and in response to ACTH stimulation including pregnenolone, 17-hydroxypregnenolone, dehydroepiandrosterone (DHEA), androstenedione, 11-deoxycortisol, and possibly cortisol.

Estradiol levels are constantly in the early to mid follicular range without the normal mid-cycle increases [**29**,**30**]. Estrone levels are increased [**29**] because of extraglandular aromatization of increased circulating androstenedione levels [**31**]. The decreased SHBG levels typical of PCOs result in increased non-SHBG bound or bioavailable estradiol as well as T levels [**32**-**34**].

Rates of abnormal range of androstenedione, LH/ FSH and LH in PCOs were significantly higher than those in the other anovulation (P < 0.01). (BMI) and hyperlipidemia in the PCOs group.

Abnormal LH/ FSH ratio is the main issue in the continuation of anovulatory state in PCOs subjects.

Increased LH and decreased or normal FSH are due to (a) GnRH pulsatile secretion, i.e. at hypothalamic level. (b) high estrogen environment, i.e., at pituitary level [**16**]. Clinically intense androgenization due to excess androgen production is observed in PCOs. Hyperandrogenemia induces the increase in testosterone, androstenedione, dehydroepiandrosterone (DHEA), DHEA-S, 17-hydroxyprogesterone and estrone (E1) (excess androgen converted to E1 by peripheral fat). The decrease in the sex hormone binding protein in the liver, increase in insulin response in the ovary and the effect of high LH, induce the increase in androgen secretion in the ovary. After that, follicle growth and maturation are suppressed.

However, gonadotropin levels have never been included in any of the diagnostic criteria for PCOs because the characteristic derangements can escape detection on random blood samples because of the pulsatile nature of LH release. 
